# *Ulmus macrocarpa* Hance modulates lipid metabolism in hyperlipidemia via activation of AMPK pathway

**DOI:** 10.1371/journal.pone.0217112

**Published:** 2019-05-23

**Authors:** Hye-Ju Han, Xinjie Song, Dhananjay Yadav, Mi Sun Hwang, Joo Hee Lee, Chang Hoon Lee, Tae Hee Kim, Jeong Jun Lee, Jungkee Kwon

**Affiliations:** 1 Department of Laboratory Animal Medicine, College of Veterinary Medicine, Chonbuk National University, Iksan, Republic of Korea; 2 Department of Food Science and Technology, Yeungnam University, Gyeongsan-si, Gyeongsangbuk-do, Republic of Korea; 3 Department of Medical Biotechnology, Yeungnam University, Gyeongsan-si, Gyeongsangbuk-do, Republic of Korea; 4 Korea Nanotechnology Center and Center for Anti-Aging Industry, Pusan National University, Geumjeong-gu, Busan, Republic of Korea; 5 Naturetech Co. Ltd., Chopyeong-myeon, Jincheon-gun, Chungbuk, Republic of Korea; Dongguk University, REPUBLIC OF KOREA

## Abstract

*Ulmus macrocarpa* Hance as an oriental medicinal plant has shown enormous potential for the treatment of several metabolic disorders in Korea. Hyperlipidemia, which is characterized by the excess accumulation of lipid contents in the bloodstream, may lead to several cardiovascular diseases. Therefore, in this study, anti-hyperlipidemic potential of *U*. *macrocarpa* water extract (UME) was examined *in vitro* and *in vivo* using HepG2 cells and experimental rats, respectively. The hyperlipidemia in experimental rats was induced by the high-cholesterol diet (HCD) followed by oral administration of various concentrations (25, 50 and 100 mg/kg) of UME for 6 weeks. As a result, the UME significantly improved the biochemical parameters such as increased the level of triglyceride, total cholesterol, and low-density lipoprotein cholesterol as well as reduced the high-density lipoprotein cholesterol in the HCD-fed rats. In addition, UME also prevented lipid accumulation through regulating AMPK activity and lipid metabolism proteins (ACC, SREBP1 and HMGCR) in the HCD-fed rats as compared to the controls. Moreover, similar pattern of gene expression levels was confirmed in oleic acid (OA)-treated HepG2 cells. Taken together, our results indicate that UME prevents hyperlipidemia via activating the AMPK pathway and regulates lipid metabolism. Thus, based on the above findings, it is estimated that UME could be a potential therapeutic agent for preventing the hyperlipidemia.

## Introduction

Hyperlipidemia is known as an abnormal state of lipid metabolism, which is characterized by imbalanced levels of lipid content specifically, increased levels of low-density lipoprotein cholesterol (LDL), total blood cholesterol (TC), and triglyceride (TG) and along with decreased levels of high-density lipoprotein cholesterol (HDL) [1,2]. Moreover, hyperlipidemia places patients at high risk for the development of cardiovascular disease (CVD), including myocardial infarction and stroke [3,4].

Adenosine monophosphate-activated protein kinase (AMPK), a central regulator of cellular energy [5], plays a dramatic role in the regulation of lipid metabolism [6]. The activation of AMPK decreases fatty acid levels by the phosphorylation of a critical enzyme, acetyl-CoA carboxylase (ACC) that regulates the oxidation and biosynthesis of fatty acids. Also, activation of AMPK reduces the levels of TC by inhibiting the enzymatic activity of HMG-CoA reductase (HMGCR), the rate-limiting enzyme of cholesterol biosynthesis [7]. Enormous amounts of research have shown that AMPK decreases the blood TG level and attenuates hepatic lipid accumulation in mice with high-fat diet-induced obesity [8,9]. Therefore, AMPK is a potential target of anti-adipogenic and anti-hyperlipidemia agents.

*Ulmus macrocarpa* Hance has been used as an oriental medicinal plant for decades as a traditional treatment for gastric ulcers, gastritis, bacterial infections, edema and infiammation in South Korea [10,11]. However, a few studies have evaluated that *U*. *macrocarpa* possesses a significant amount of pharmacological potential such as anti-cancer, anti-allergic, anti-oxidant, anti-inflammatory, and anti-platelet activities [12], the reports on its anti-hyperlipidemic potential are scarce with an underlying mechanism.

Therefore, in the present study, the anti-hyperlipidemic effects of the water bark extract of *U*. *macrocarpa* (UME) on lipid accumulation in HepG2 cells were investigated by measuring the expression levels of lipid metabolism genes and Oil Red O staining. Moreover, *in vivo* potential of the UME was assessed using a high-cholesterol diet (HCD)-fed rats to verify the anti-hyperlipidemia effect of UME.

## Materials and methods

### Preparation of the water extract of *U*. *macrocarpa* (UME)

*Ulmus macrocarpa* Hance bark samples were purchased from JND. Inc. (Busan, Korea). A 100 kg of bark sample was extracted with water at 95°C for 6 h with a mixing ratio of 1:10. During the extraction process, viscozyme (0.4%) was added, and the extract was reacted at 50°C for 2 h. The enzyme reaction was terminated by incubating the sample at 100°C for 10 min, and the extract was filtered using a 1 μm filter and concentrated using a vacuum rotary evaporator. The final brix value reached approximately 16.0. The extract was freeze-dried after sterilization at 90°C for 30 min.

### Cell culture

HepG2 cells were purchased from American Type Culture Collection (USA). The cells were cultured in humid condition at 370°C under 5% CO_2_ environment in DMEM medium (Gibco BRL, NY, USA) supplemented with fetal bovine serum (10%), penicillin G (100 U/mL), and streptomycin (100 mg/mL). Cell numbers were adjusted by counting the cells under hemocytometer. An AMPK inhibitor (compound C) and oleic acid (OA) were procured from Sigma (St. Louis, MO, USA).

### Cell viability assay

The cytotoxicity of the water extract of *U*. *macrocarpa* (UME) was measured via MTT [3-(4,5-dimethylthiazol-2-yl)-2,5-diphenyltetrazolium bromide] assay. The cells were cultured in 96-well plates, and treated with various concentrations of UME (25, 50, 100 and 200 μg/mL) followed by incubation. After that old medium was discarded and fresh medium containing 0.5 mg/mL MTT was added to each well, and further incubated at 37°C for 4 h. Following this, dimethyl sulfoxide (200 μL) was added to each well to dissolve the formed crystals of formazan product after removing the medium from each well. Absorbance was then measured at 540 nm to quantify the reduction ability of UME within the cells from MTT to formazan using a PowerWave2 Multi-plate Reader (Bio-Tek Instruments, Winooski, VT, USA). The cytotoxicity was calculated using the following equation:

Cellviability(%)=SampleOD/BlankOD×100

### Oil red O staining

Oil Red O staining was performed using the commercial staining kit obtained from Lifeline Cell Technology, USA. Oil red O-stained cell cultures were photographed at the magnification of 200X using an A1 microscope (Carl Zeiss, Jena, Germany). After taking the photograph, the Oil Red O stained culture dye retained in the cells was eluted with isopropanol. Quantification of the retained dye was performed by measuring the absorbance at 540 nm, using a Multi-plate Reader (Bio-Tek, USA).

### Preparation of RNA and real-time (RT)-PCR

Total RNA from HepG2 cells was extracted using the TRIzol RNA extraction kit following the kit manual (Invitrogen, CA, USA) and the aqueous layer of the supernatant was collected. A 2 μg of total RNA with reverse transcriptase (200 units), and oligo (dT)_12-18_ primers (25 μg/ml) was used for performing the reverse transcription. 5 ng cDNA was amplified for RT-PCR using SYBR Green PCR Master Mix with 0.33 μM each primer (forward and reverse) on the ABI StepOnePlus RT-PCR system (Applied Biosystems) with published techniques [13]. Designing of primers for RT-PCR was performed using the Primer Express Software v3.1 (Applied Biosystems) and synthesized at IDT Corporation. The target genes and primers used are shown in Table 1. All experiments were set in 3 replicates, and quantification of the data was based on the mean values calculated. StepOne Software v2.3 from Applied Biosystems, was employed for the analysis following the manufacturer's instructions. A housekeeping gene GAPDH was employed for sample normalization. Results for treatment cells were presented as the increase in the relative fold and compared with those of control HepG2 cells.

**Table 1:** Primers used for analysis of the expression of AMPK pathway and lipid metabolism-related genes.

Gene	5' 5'	Forward primer Reverse primer	3' 3'
AMPK	TCTCAGGAGGAGAGCTATTTGATTATATC TGGAACAGACGCCGACTTTC		
SREBP1	GCTGTCCACAAAAGCAAATCTCT GTCAGTGTGTCCTCCACCTCAGT		
ACC1	GGATCCGGCGCCTTACTT CTCCGATCCACCTCATAGTTGAC		
HMGCR	TTCTGACAATAACACGATGCATAGC AAGGCCAGCAATACCCAAAA		
GAPDH	CTGCCCCCTCTGCTGATG AGGAGGCARTGCTGATGATCTT		

### *In vivo* experimental design

Hyperlipidemia in the experimental rats was induced by HCD (#D12336, Research Diets Inc., NJ, USA). The normal group was fed a control diet (CD) (#D12337, Research Diets Inc., NJ, USA). Atorvastatin and omega-3 used as positive controls were obtained from Sigma. Five-weeks-old male Sprague Dawley rats (n = 10) were purchased for animal studies from Damul Science Ltd. (Daejeon, Korea) and were allowed to adaptation for a week before the animal studies. The experimental rats were randomly divided into seven groups which were as follows: Normal group (CD, alone), Control group (HCD, alone), UME groups (HCD and each UME 25, 50 and 100 mg/kg), ATO group (HCD and atorvastatin 1.023 mg/kg), and O3 group (HCD and omega-3 170 mg/kg) for 6 weeks. All experimental rats were allowed to access water and food with UME. The food intake of experimental rats was measured daily, whereas the body weights were measured weekly after the beginning of UME treatment. Rats were housed under pathogen-free conditions in micro-isolator cages with a rotation of 12-h light/dark cycle. Blood samples collected from the inferior vena cava were used to analyze the serum biomarkers. For histology and immunoblotting analyses, lever samples were harvested from the experimental rats, weighed, and processed before blood collection. All collected samples were stored at −80°C until further analysis. International standard for care and use of experimental animals was followed. The animal study was specifically approved (Approval number: CBNU 2018–068) by the institutional committee for the care and use of animals of the Chonbuk National University, Jeonju, Korea.

### Biochemical analysis

The aspartate aminotransferase (AST) and alanine aminotransferase (ALT) assays were performed according to the kit manufacturer’s instructions (Asan Pharm, Seoul, Korea). TC, HDL-c and LDL-c were measured according to the instructions of kit manufacturer’s (Abcam, Cambridge, MA, UK). TG was measured according to the kit manufacturer’s instructions (Cayman Chemical, Ann Arbor, MI).

### Western blot analysis

A lysis buffer [NaCl (140 mM), Tris–HCl (25 mM; pH 7.4), and 1% NP-40] and freshly prepared protease inhibitor cocktail were used for harvesting the liver tissue extracts from the experimental rats. Sodium dodecyl sulfate polyacrylamide gel electrophoresis of proteins was performed on a 10–15% agarose gel and transferred to PVDF membranes (BioRad, Hercules, CA). After blocking in 5% skim milk in PBS, the membrane was incubated with each primary antibody for SREBP1 and HMGCR (Abcam, Cambridge, MA, UK) and AMPK, p-AMPK, ACC, p-ACC and β-actin (Cell Signaling, Danvers, MA, USA), and then diluted 1:1000 with 1% skim milk in PBS at 4°C overnight. Blots were then incubated with peroxidase-conjugated secondary antibody goat anti-rabbit IgG (Millipore, Temecula, CA, USA) diluted 1:10,000 at room temperature for 1 h. Immunoreactive bands were detected by a Super Signal West Dura Extended Duration Substrate (Thermo, CA, USA), following the manufacturer's instructions. A Chemi Imager Analyzing System (Alpha Innotech, CA) was used for the densitometric analysis directly from the blotted membrane.

### Histopathology

A 10% buffered solution of formalin was used for fixing the liver tissue samples for 24 h followed by embedding them in paraffin, and the tissues were chopped at 4−5 μm. Xylene solution was used for de-paraffinizing of the tissue sections followed by dehydration using the graded concentrations of alcohol. Staining of the tissue sections was then performed using a hematoxylin and eosin (H&E) stain. Histology of the tissue sections was observed under a conventional light microscope. Images of H&E stained tissue sections were then captured and the area of the stained regions was pixel quantified by the Image Pro-analysis software.

### Statistical analysis

All sets of experiments were executed at least in three replicates. Data values were presented as mean ± standard error. Statistical differences between the groups were determined by one-way ANOVA and Student's t-test. P values < 0.05 were considered statistically significant.

## Results

### Effects of UME on cell viability and lipid accumulation in HepG2 cells

**Fig 1:**
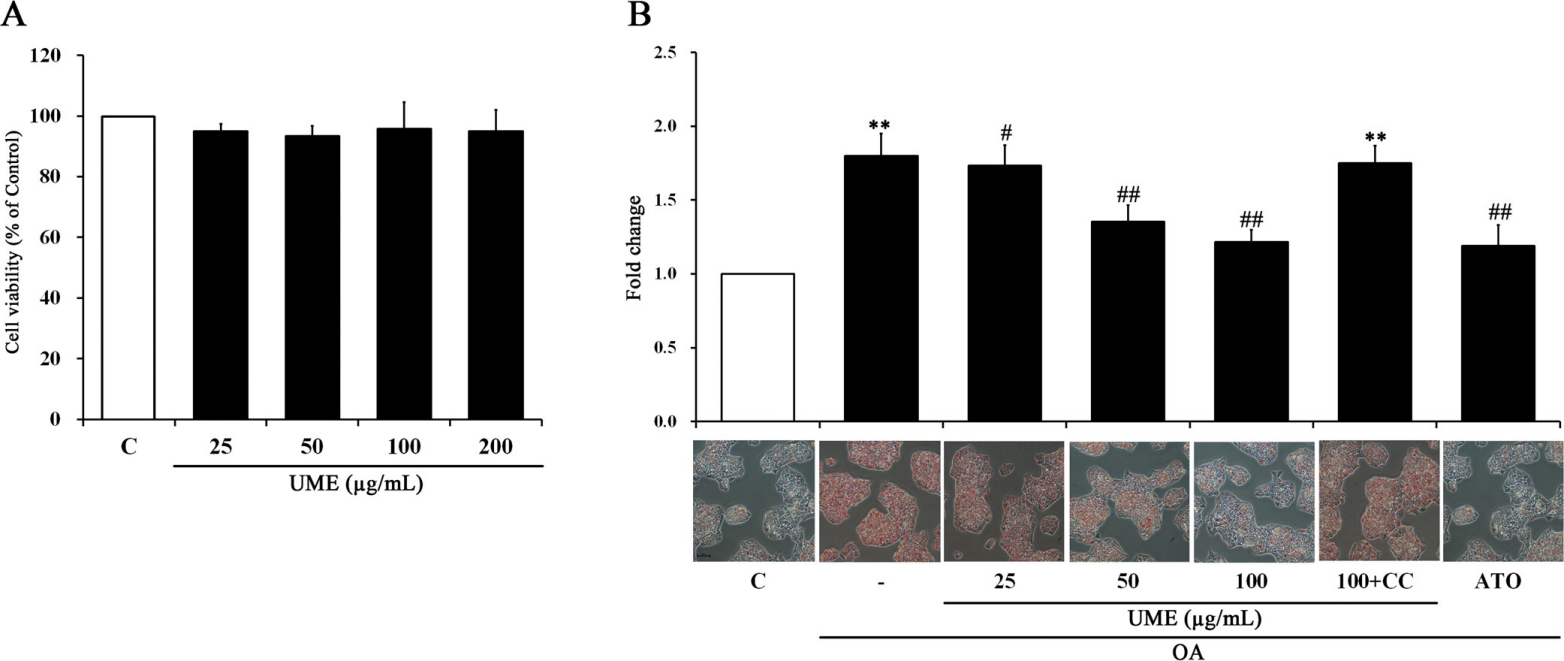
Cytotoxicity and lipid accumulation in UME-treated HepG2 cells. (A) Cell viability was determined with an MTT assay. HepG2 cells were treated with various concentrations of UME (25, 50, 100, and 200 μg/mL). (B) Intracellular lipid droplets in HepG2 cells were stained with oil red O and observed at 200× magnification. HepG2 cells were treated with compound C (10 μM) for 1h and then incubated with or without UME (25, 50 and 100 μg/mL) and ATO (10 μM) for an additional 1 h. Cells were then incubated with OA for 1 h. Lipid droplets were quantified by measuring the resultant absorbance at 540 nm. Data are expressed as the mean ± SEM (n = 10). **p < 0.01, compared to the control. #p < 0.05, compared to the OA-only treated group. ##p < 0.01, compared to the OA-only treated group. UME: *Ulmus macrocarpa* Hance extracts, CC: compound C, ATO: atorvastatin, OA: oleic acid.

The cytotoxicity and effect of UME on lipid accumulation were analyzed by the MTT assay and Oil Red O staining, respectively. As a result, UME-treated cells did not exhibit significant cell death (Fig 1A). As shown in Fig 1B, intracellular lipid quantification results revealed that UME at the treatment concentrations of 50 and 100 μg/mL dose-dependently attenuated OA-induced lipid accumulation compared to the OA-alone group. Treatment with compound C, an AMPK inhibitor, led to reversed results compared to UME treatment. These results suggested that UME does not exhibit cytotoxicity in HepG2 cells and reduces lipid accumulation in OA-treated HepG2 cells (S1 File).

### Effect of UME on lipid metabolism genes involved in AMPK activation in OA-treated HepG2 cells

**Fig 2:**
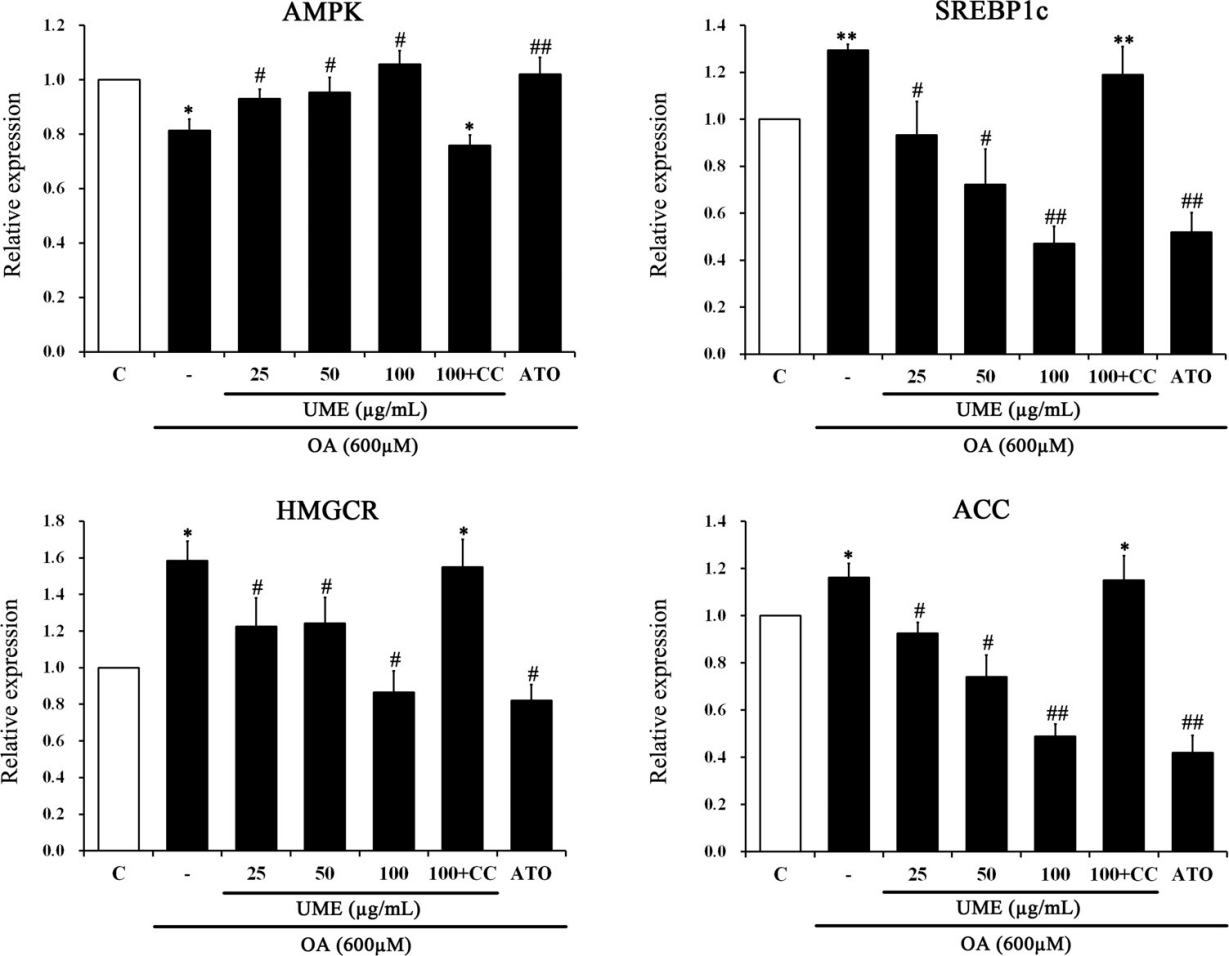
The expression levels of the lipogenic genes AMPK, SREBP1c, HMGCR, and ACC in HepG2 cells were examined by real-time RT-PCR. Data are expressed as the mean ± SEM (n = 10). *p < 0.05, compared to the control. **p < 0.01, compared to the control. #p < 0.05, compared to the OA-only treated group. ##p < 0.01, compared to the OA-only treated group. UME: *Ulmus macrocarpa* Hance extracts, CC: compound C, ATO: atorvastatin, OA: oleic acid.

To determine the effect of UME on lipid metabolism gene regulation, we measured AMPK, ACC, SREBP1 and HMGCR gene expression levels in HepG2 cells by real-time RT-PCR after treatment with OA. As shown in Fig 2, the OA-alone group showed significantly decreased expression of the AMPK gene and increased expression of the SREBP1c, HMGCR and ACC genes compared to the control, whereas UME treatment significantly reversed the OA-alone gene expression pattern. In addition, the UME groups and the ATO group showed similar effects. Compound C treatment had a reversed gene expression pattern compared to UME treatment. These results indicate that UME activates the AMPK pathway and regulates lipid metabolism in OA-treated HepG2 cells (S1 File).

### Effects of UME on body weight and liver weight in HCD-fed rats

As shown in Table 2, body weight and feed intake did not differ significantly between groups. The HCD-alone (control) group had a slightly increased feed efficiency ratio (FER) compared to the normal group; however, these changes did not differ significantly compared with the other groups. We found that the control group had significantly increased liver weight per body weight compared to the normal. The UME groups had significantly decreased liver weight per body weight in a dose-dependent fashion as compared to the control. The O3 group liver weight per body weight was also significantly decreased. Interestingly, the UME 50 μg/mL and UME 100 μg/mL groups showed a greater decrease in liver weight per body weight compared with ATO, which is an accepted treatment for hyperlipidemia.

**Table 2:** Body weight gain, feed intake, feed efficiency ratio (FER) and liver weight/body weight in rats fed HCD with UME.

Groups	Body weight gain (g/day)	Feed intake (g/day)	FER	Liver weight/ Body weight (%)
NOR	5.66 ± 2.38	20.64 ± 0.95	0.27 ± 0.11	2.91 ± 0.21
CON	5.67 ± 1.97	18.06 ± 1.11	0.31 ± 0.10*	5.25 ± 0.44***
UME25	4.95 ± 2.28	16.94 ± 1.22	0.29 ± 0.12	4.74 ± 0.27
UME50	5.68 ± 2.23	18.04 ± 1.05	0.31 ± 0.11	4.69 ± 0.20 #
UME100	5.41 ± 2.09	18.55 ± 1.06	0.29 ± 0.11	4.67 ± 0.26 #
ATO	5.49 ± 2.52	18.56 ± 1.19	0.29 ± 0.12	4.78 ± 0.28
O3	5.29 ± 2.20	17.04 ± 0.73	0.31 ± 0.12	4.65 ± 0.21 #

FER: feed efficiency ratio = body weight gain / feed intake.

*p < 0.05, compared to normal.

***p < 0.001, compared to normal.

#p < 0.05, compared to the control. NOR: normal, CON: high-cholesterol diet (HCD), UME 25–100: HCD + UME (25, 50, 100 mg/kg), ATO: HCD + atorvastatin, O3: HCD + omega-3.

### Effects of UME on hepatic toxicity in HCD-fed rats

**Fig 3:**
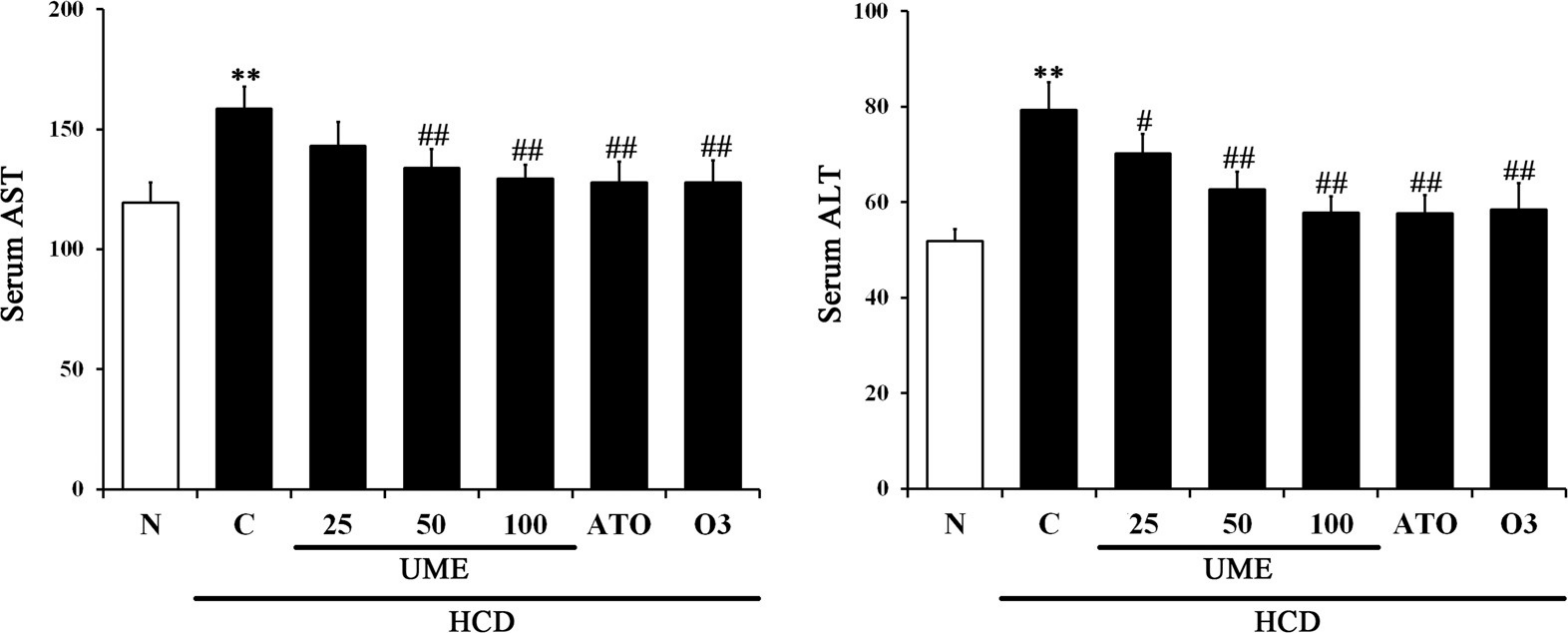
Levels of AST and ALT in UME-treated HCD-fed rats. The levels of ALT and AST in serum were measured according to the kit manufacturer’s instructions. Data are expressed as the mean ± SEM (n = 10). AST: aspartate aminotransferase, ALT: alanine aminotransferase. **p < 0.01, compared to normal. #p < 0.05, compared to the control. ##p < 0.01, compared to the control. N: normal, C: high-cholesterol diet (HCD), UME 25–100: HCD + UME (25, 50, 100 mg/kg), ATO: HCD + atorvastatin, O3: HCD + omega-3.

The levels of ALT and AST as key biomarkers of liver damage were analyzed (refer Materials and Methods section). There was a significant increase in the AST and ALT levels of serum in the control, whereas UME treatment significantly decreased serum AST and ALT levels in a dose-dependent fashion (Fig 3; S1 File). In addition, the ATO and O3 groups had significantly decreased serum AST and ALT levels compared to the control. Interestingly, the UME, ATO and O3 groups experienced similar effects. These results indicate that UME reduced liver toxicity in hyperlipidemic experimental animals.

### Effects of UME on serum lipid profile in HCD-fed rats

**Fig 4:**
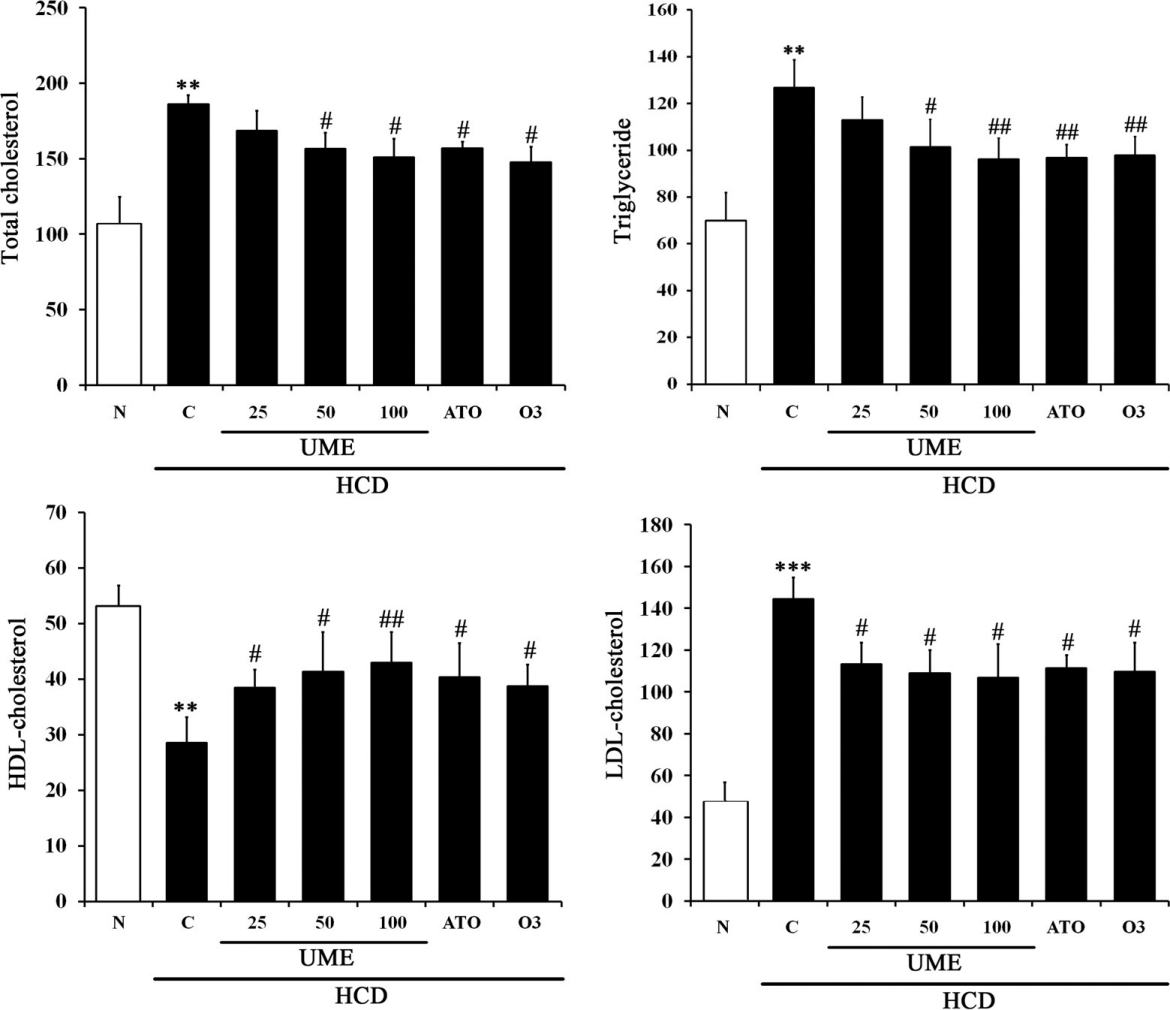
Lipid biomarkers in the serum of UME-treated HCD-fed rats. (A-D) Serum concentrations of total cholesterol, triglyceride, HDL cholesterol and LDL cholesterol were measured by a commercial analysis kit. Data are expressed as the mean ± SEM (n = 10). **p < 0.01, compared to normal. ***p < 0.001, compared to normal. #p < 0.05, compared to the control. ##p < 0.01, compared to the control. N: normal, C: high-cholesterol diet (HCD), UME 25–100: HCD + UME (25, 50, 100 mg/kg), ATO: HCD + atorvastatin, O3: HCD + omega-3.

To determine the effect of UME on lipid metabolism regulation, we measured TC, TG, HDL-c and LDL-c in serum. As shown in Fig 4, the control group showed significantly increased levels of TC, TG, and LDL-c, but not HDL-c, compared to normal. The UME groups showed significantly decreased levels of TC, TG, and LDL-c, but not serum HDL-c, compared to control. Interestingly, the UME, ATO and O3 groups had similar effects in serum HDL and LDL levels (S1 File). These results strongly indicate that UME prevents hyperlipidemia by improving lipid accumulation.

### Effects of UME on cardiac risk factor (CRF) and atherogenic index (AI) in HCD-fed rats

As shown in Table 3, the control group had significantly increased CRF and AI levels compared to the normal group. On the other hand, UME treatment significantly decreased CRF and AI levels dose-dependently when compared with the control group. In addition, the ATO and O3 groups had significantly decreased the levels of CRF and AI as compared to the control. These results suggest that UME treatment reduces cardiovascular risk factors and atherosclerotic index, which increase with a HCD.

**Table 3:** Cardiac risk factor (CRF) and atherogenic index (AI) in the serum of rats.

Groups	CRF	AI
NOR	2.11 ± 0.29	1.18 ± 0.25
CON	6.52 ± 1.12 **	5.13 ± 0.62 ***
UME25	4.75 ± 0.62 #	3.55 ± 0.61 #
UME50	4.26 ± 0.54 #	3.00 ± 0.52 #
UME100	4.01 ± 0.73 #	2.86 ± 0.75 #
ATO	4.08 ± 0.61 #	3.08 ± 0.62 #
O3	4.05 ± 0.56 #	3.05 ± 0.53 #

CRF: cardiac risk factor = TC/HDL-c, AI: atherogenic index = (TC-HDL-c)/HDL-c.

**p < 0.01, compared to normal.

***p < 0.001, compared to normal.

#p < 0.05, compared to the control. NOR: normal, CON: high-cholesterol diet (HCD), UME 25–100: HCD + UME (25, 50, 100 mg/kg), ATO: HCD + atorvastatin, O3: HCD + omega-3

### Effects of UME on TC and TG levels in the livers of HCD-fed rats

**Fig 5:**
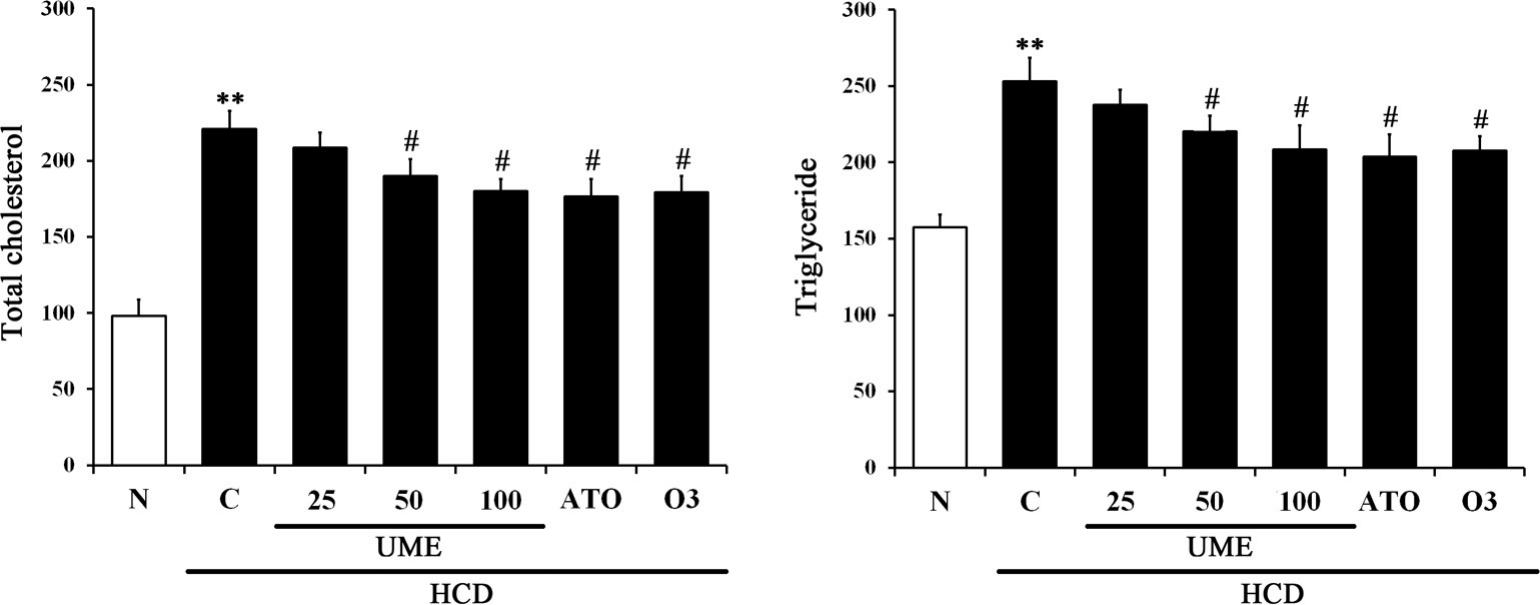
Levels of total cholesterol and triglyceride in the livers of UME-treated HCD-fed rats. Liver concentrations of total cholesterol and triglyceride were measured by a commercial analysis kit. Data are expressed as the mean ± SEM (n = 10). **p < 0.01, compared to normal. #p < 0.05, compared to the control. N: normal, C: high-cholesterol diet (HCD), UME 25–100: HCD + UME (25, 50, 100 mg/kg), ATO: HCD + atorvastatin, O3: HCD + omega-3.

To determine the effect of UME on the lipid content of liver tissue, the levels of TC and TG in the liver tissue of each experimental group were measured. As shown in Fig 5 and S1 File, the control group had significantly increased TC and TG levels in liver tissue compared to the normal. As a result, UME treatment dose-dependently decreased the levels of TC and TG in liver tissues when compared with the control. Moreover, the ATO and O3 groups had significantly decreased levels of TC and TG in liver tissues. In particular, the UME group had similar levels to the ATO and O3 groups. These results showed that UME lowers the lipid content in liver tissue of HCD-induced hyperlipidemic rats.

### Effects of UME on AMPK phosphorylation and lipid metabolism in HCD-fed rats

**Fig 6:**
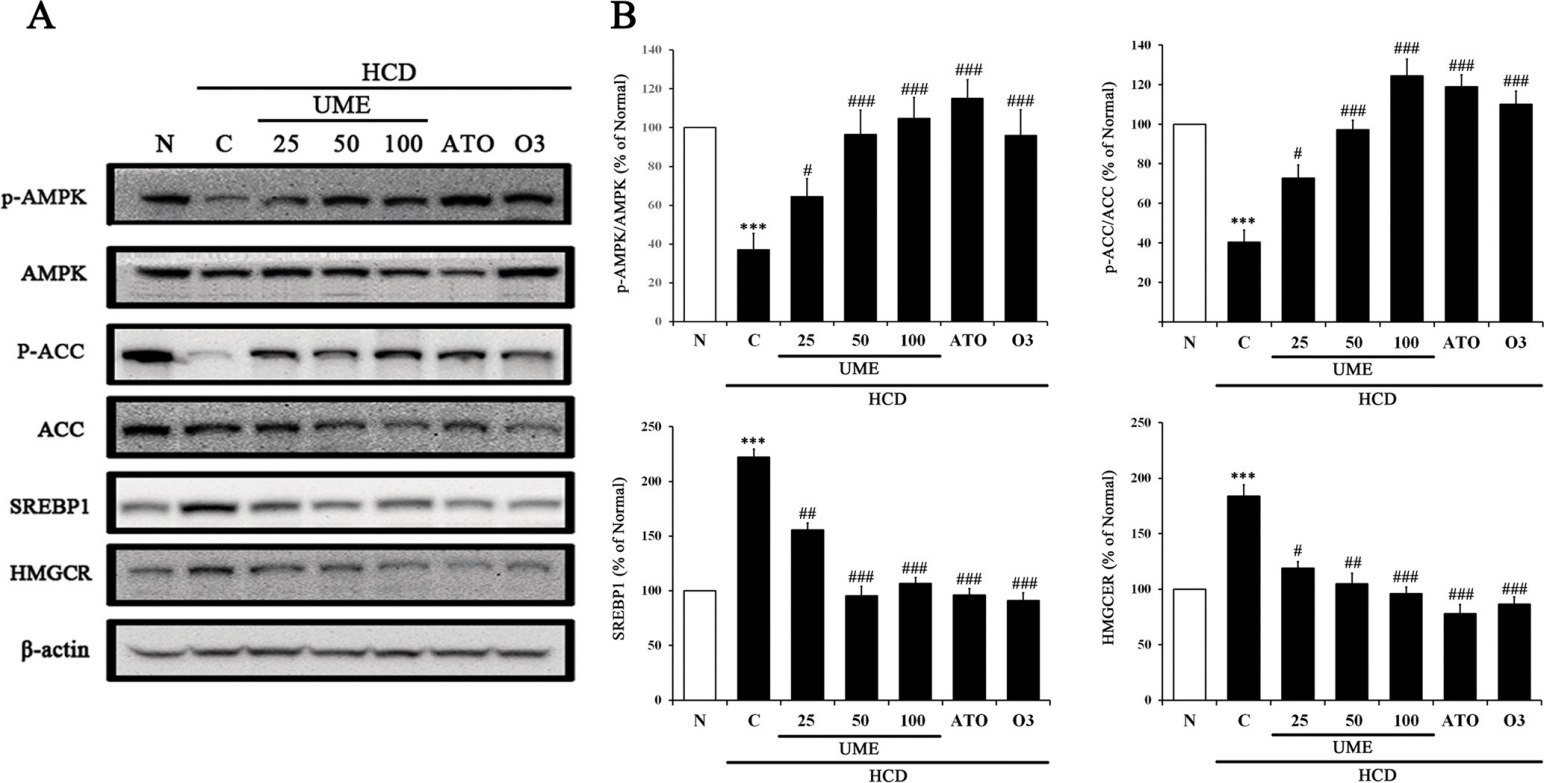
Activation of AMPK pathway in the liver of UME-treated HCD-fed rats. (A) The protein expression levels of p-AMPK, AMPK, p-ACC, ACC, SREBP1 and HMGCR in liver tissue were measured by western blot. (B) The bar graphs indicate the average p-AMPK/AMPK, p-ACC/ACC, SREBP1, and HMGCR activity. Data are expressed as the mean ± SEM (n = 10). ***p < 0.001, compared to normal. #p < 0.05, compared to the control. ##p < 0.01, compared to the control. ###p < 0.001, compared to the control. N: normal, C: high-cholesterol diet (HCD), UME 25–100: HCD + UME (25, 50, 100 mg/kg), ATO: HCD + atorvastatin, O3: HCD + omega-3.

We further confirmed the association of UME with the AMPK pathway and lipid metabolism by examining the AMPK, ACC, SREBP1, and HMGCR proteins. As shown in Fig 6 and S1 File, the control group had significantly decreased expression of phosphorylated AMPK (p-AMPK) and phosphorylated ACC (p-ACC), and increased expression of SREBP1 and HMGCR, compared to the normal. The UME treatment groups had significantly increased expression of p-AMPK and p-ACC in a dose-dependent manner, and decreased expression of SREBP1 and HMGCR compared to the control. Interestingly, the UME groups showed a similar protein expression pattern to the ATO and O3 groups. These results indicate that UME activates the AMPK pathway and regulates lipid metabolism in HCD-induced hyperlipidemic animals.

### Effect of UME on lipid accumulation in the liver tissues of HCD-fed rats

**Fig 7:**
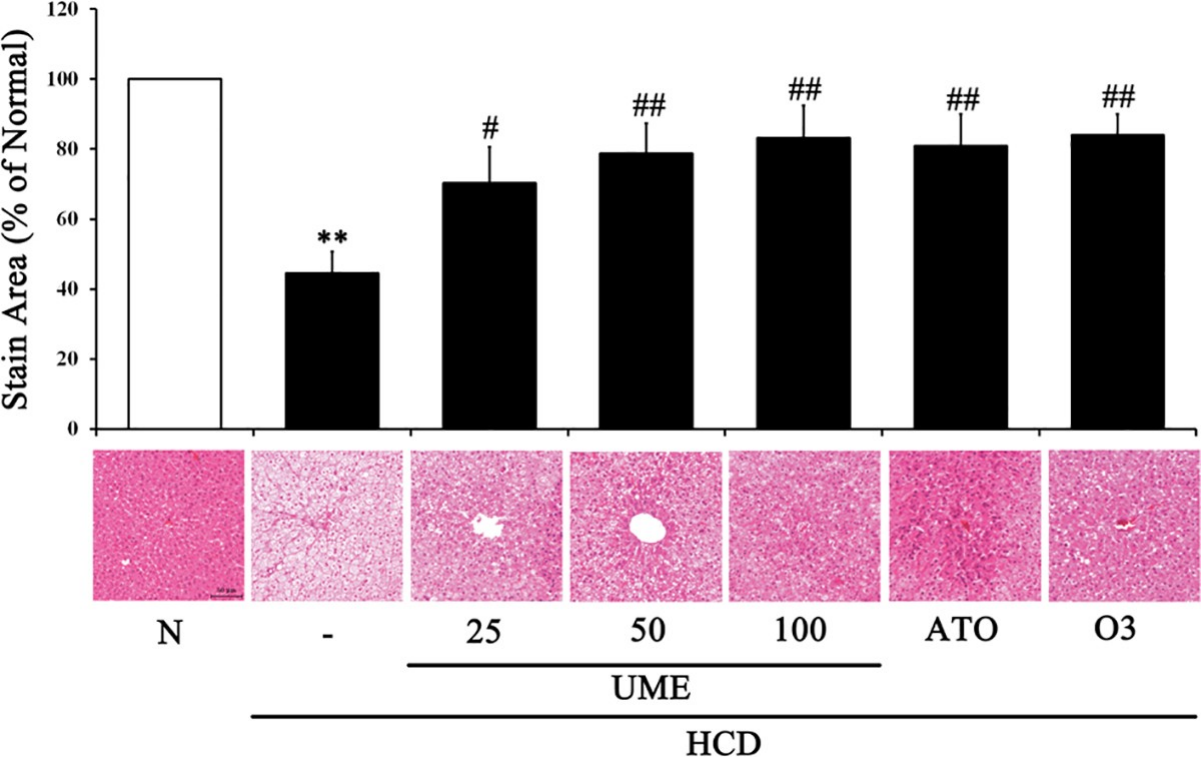
Hepatic lipid accumulation in UME-treated HCD-fed rats. H&E staining (magnification 200×) was quantified by determining the stained area, in pixels, in each image using the Image Pro analysis program. Data are expressed as the mean ± SEM (n = 10). **p < 0.01, compared to normal. #p < 0.05, compared to the control. ##p < 0.01, compared to the control. N: normal, C: high-cholesterol diet (HCD), UME 25–100: HCD + UME (25, 50, 100 mg/kg), ATO: HCD + atorvastatin, O3: HCD + omega-3.

To identify the effect of UME on HCD-induced lipid accumulation in experimental animals, tissue samples from liver and aorta were prepared from the treatment and control groups followed by H&E staining. The results of H&E staining demonstrated that lipid droplet appeared as small vacuoles within the liver cells of control group (Fig 7; S1 File). These histological abnormalities were reduced in the liver tissues by the treatment with UME in a dose-dependent fashion (Fig 7). These results suggest that UME improved the lipid accumulation in HCD-induced hyperlipidemic rats.

## Discussion

Hyperlipidemia is a key risk factor for cardiovascular diseases, which are among the leading causes of mortality throughout the globe [14]. Common characterizations of hyperlipidemia include enhanced levels of TC, TG, and LDL and decreased HDL. These are known to be critical factors in heart diseases, including atherosclerosis and coronary heart disease [15–17]. Previous studies have reported on various statin drugs such as fluvastatin and atorvastatin, which are used to induce hypolipidemia or to prevent hyperlipidemia. Atorvastatin acts by inhibiting HMGCR, the rate-limiting enzyme of the cholesterol biosynthetic pathway [18]. Previous studies have shown that omega-3 reduces TG in hyperlipidemic individuals and may extend to normolipidemic populations [19,20]. Thus, we used these drugs and functional foods as positive controls in the *in vivo* experiments.

AMP-activated protein kinase (AMPK) is well known to play a critical role in controlling lipid metabolism, glucose homeostasis and insulin sensitivity. AMPK is a serine/threonine kinase that regulates lipid metabolism by phosphorylating and inactivating its well-recognized downstream target acetyl CoA carboxylase (ACC). AMPK promotes fatty acid oxidation and inhibits hepatic lipid accumulation upon its activation [21]. Sterol regulatory element binding protein-1 (SREBP-1), an important transcription factor that regulates the expression of ACC, stimulates a number of adipogenic enzymes involved in the synthesis of hepatic fatty acids and regulates the lipogenic process by activating genes involved in fatty acid and TG synthesis [22]. Therefore, HMGCR and ACC, two enzymes involved in cholesterol and lipogenesis, are regarded as the main targets of AMPK and are phosphorylated by enzymes, leading to cholesterol and TG synthesis inhibition followed by inhibition of hepatic lipid accumulation by AMPK. The UME treatment significantly elevated the expression of AMPK and decreased levels of SREBP1c, HMGCR and ACC in OA-treated HepG2 cells (Fig 2). In addition, treatment with compound C, an AMPK inhibitor, reversed gene expression compared to UME treatment. These results advocate that UME antagonizes hepatic lipid accumulation induced by OA *via* activating the AMPK pathway.

Following the results of *in vitro* assays, we performed an *in vivo* experimental study using HCD-induced hyperlipidemic rats. All the experimental rats employed throughout the *in vivo* studies were apparently healthy and showed no sign of any pathological abnormalities. Changes in body weight, feed intake, FER, and liver weight per body weight during the experimental period are shown in Table 2. No significant differences were observed among the FER groups, except in the control group. However, liver weight per body weight was significantly increased in the control group fed HCD. The accumulation of cholesterol and triglycerides by HCD increases liver weight. UME treatment significantly decreased liver weight per body weight in a dose-dependent fashion when compared with the control. These results showed that UME decreased the weight of liver tissue per body weight via inhibited liver lipid accumulation. Previous studies have shown that increased levels of LDL cholesterol are correlated with an increased incidence of coronary artery diseases [23,24]. We found that daily oral administration of UME in HCD-induced hyperlipidemic rats reduced serum TC, TG and LDL-c compared to the control group. In addition, oral administration of UME increased serum HDL-c (Fig 4). It also decreased the levels of CRF and AI in a dose-dependent manner compared to the control group (Table 3). These results suggest that UME improves the risk of arteriosclerosis and circulatory system disease through inhibiting blood lipid accumulation.

As shown in Fig 6, UME treatment increased the expression levels of p-AMPK and p-ACC and decreased the levels of SREBP1 and HMGCR in liver tissues. Interestingly, the UME, ATO and O3 groups showed similar protein expression patterns. These results suggest that UME prevents hyperlipidemia via activation of AMPK. Similarly, Lee et al. [25] also reported that plant-based extract was able to suppress SREBP-1 and HMGCR expression levels and significantly decreased TG content and lipid accumulation in liver tissues by activating the AMPK pathway *in vitro* and *in vivo*. Based on the consistent results of our studies, we found that UME treatment resulted in the significant reduction of lipid accumulation in the liver tissues of the experimental rats compared to the control (Fig 7).

**Fig 8:**
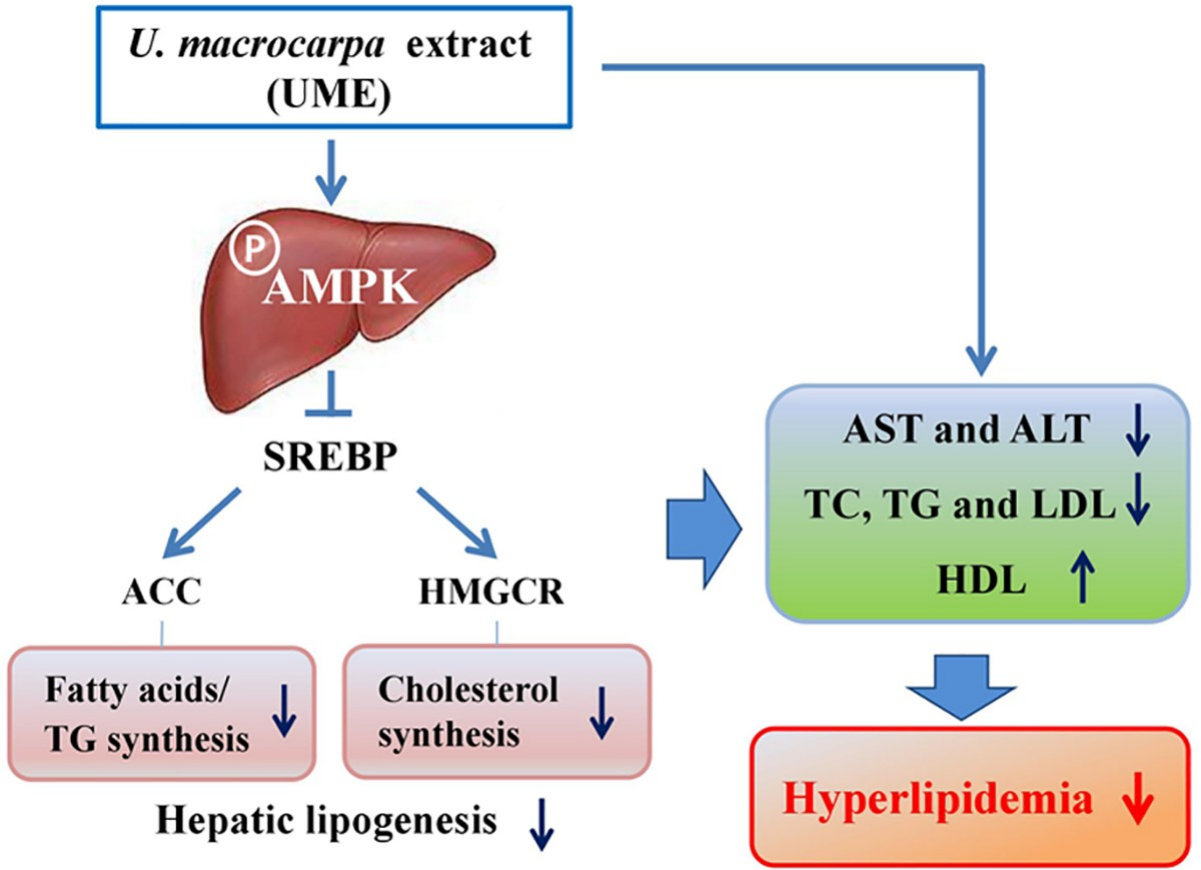
Predictable mechanism in hepatic tissues of UME-treated HCD-fed rats. **UME prevented hyperlipidemia by up-regulating the AMPK pathway and regulation of lipid metabolism.** SREBP1: sterol regulatory element binding protein-1, ACC: acetyl CoA carboxylase, HMGCR: 3-hydroxy-3-methylglutaryl-CoA reductase, AST: aspartate aminotransferase, ALT: alanine aminotransferase, TC: total cholesterol, TG: triglyceride, LDL: low-density lipoprotein cholesterol, HDL: high-density lipoprotein cholesterol.

Collectively, these results show that UME exerts an anti-hyperlipidemia effect on OA-treated HepG2 cells and in an HCD-induced hyperlipidemia animal model. Moreover, this study showed that UME prevented hyperlipidemia *in vivo* via activation of the AMPK pathway and regulation of lipid metabolism (Fig 8). This could be a natural candidate of significant therapeutic potential. Further *in vivo* studies on specific bioactive compounds present in the UME are planned to validate its biomedicinal potential.

## Supporting information

S1 File Supporting information raw data. This file contains raw data including cell viability, expression of lipogenic genes (AMPK, SREBP1c HMGCR, and ACC), AST and ALT levels, serum lipid biomarkers, cholesterol and triglyceride levels, protein expression levels of p-AMPK AMPK, p-ACC, SREBP1, and HMGCR, and H&E staining results.
(XLSX)
